# *¡Coma*,* Muévase y Viva!*: qualitative findings from a dietary and lifestyle change intervention for Latinas in the rural desert region of Inland Southern California

**DOI:** 10.1186/s12889-025-25081-1

**Published:** 2025-11-19

**Authors:** Jacqueline Moreira, Andrea Gonzalez, Jair Chavez, Noah Baltrushes, Ann Marie Cheney

**Affiliations:** 1 School of Medicine, University of California Riverside, Riverside, CA USA; 2 Bloomberg School of Public Health, John Hopkins University, Baltimore, MD USA; 3 College of Agricultural and Natural Sciences, University of California Riverside, Riverside, CA USA; 4 Department of Social Medicine, Population and Public Health, SOM Bldg 1, SMPPH, 900 University Avenue, Riverside, CA 92521 USA

**Keywords:** Latino, Indigenous Mexican, Purépecha, Dietary and lifestyle intervention, Rural, Immigrant health

## Abstract

**Background:**

Racial-ethnic minorities experience inequities that contribute to chronic disease burden. Subpopulations of Latinos such as those living in rural communities have their own unique health needs and barriers to chronic disease management and control. Interventions culturally tailored to the distinct needs of diverse Latinos are ideal to address inequities in health. This study examined the perceptions and experiences of participants engaged in a dietary and lifestyle behavior change curriculum, ¡Coma, Muévase y Viva! a public health intervention tailored for rural Latinx immigrant populations.

**Methods:**

A qualitative evaluation, involving focus groups and observations, of the perceived effects of an adapted version of a dietary and lifestyle curriculum on health outcomes was conducted in fall/winter 2022 in Inland Southern California. The curriculum is a 10-week virtual intervention with weekly 120-minute classes featuring health education, physical activity, and cooking demonstrations.

**Results:**

A total of 20 Latina women (mean age 45 ± 23 years) participated in the study. The majority identified as Latinx/Hispanic: 80% indicated Spanish as their primary language, and 70% were originally from Mexico. Half (50%) had not completed high school, 50% reported household incomes below $25,000, and 40% were uninsured. Most (85%) were concerned about obesity with 70% meeting the criteria for obesity. Participants perceived the curriculum as increasing their knowledge and awareness of chronic disease prevention, which contributed to improvements in dietary behaviors (e.g., increased fruit and vegetable consumption, reduced intake of sugary beverages) and physical activity. These changes, observed following the intervention, resulted in self-reported weight loss as well as enhanced physical agility, confidence, and ability to manage chronic diseases. Participants were highly satisfied with the intervention, suggesting that the curriculum’s accessibility and cultural relevance contributed to its acceptability.

**Conclusions:**

The culturally tailored ¡Coma, Muévase y Viva!, intervention offers a promising curriculum to increase access to public health education and promote chronic disease prevention and management in low-income, rural immigrant populations through changes in diet and lifestyle.

**Supplementary Information:**

The online version contains supplementary material available at 10.1186/s12889-025-25081-1.

## Introduction

 Systemic inequalities in education and employment systems push racial-ethnic minority populations into neighborhoods with limited resources, including food deserts [1]. Food deserts, characterized by limited access to grocery stores and affordable fresh produce, create barriers to maintaining healthy dietary intake [1]. This is an especially salient concern in rural areas of the United States (US). Rural areas often lack healthcare services, green and recreational spaces, and public transportation, all of which figure into individual and community health outcomes [2]. Latinos, the largest racial-ethnic minority population in rural America, are highly impacted by these intersecting disadvantages [3, 4]. This complex issue contributes to a high prevalence of chronic diseases, especially diabetes, among low-income Latinos in rural communities [5].

Scientific evidence suggests that a healthy diet and physical activity are crucial to preventing diet-related chronic diseases (diabetes, cardiovascular disease, and some cancers) [6]. Diabetes is the eighth leading cause of death in the US [7], and the number of adults diagnosed with diabetes has more than doubled over the past 20 years, with Latinos being overrepresented in this group [7]. This combination of factors suggests early intervention and health education are key to preventing type 2 diabetes and other chronic diseases in this population.

Addressing barriers to diet and lifestyle change behaviors is critical to address racial and ethnic disparities in chronic disease burden. Many Latinos in rural America experience food insecurity due to employment in low-wage, contingent labor such as agriculture and residence in a rural geographic location, making it difficult to purchase fresh fruits, vegetables, and other healthy ingredients to prepare meals [8, 9]. This often leads to higher consumption of processed foods, which are usually more accessible and affordable, especially in rural areas with limited food retailers [10].

One way to address this is through culturally tailored diet and lifestyle change interventions that aim to reduce inequities in food access and increase public health knowledge about chronic disease prevention and management [11, 12]. Many community-based programs tend to focus solely on alleviating food insecurity rather than simultaneously increasing access to healthy foods and providing health education on healthy eating and regular physical activity [13]. Furthermore, very few interventions are tailored for low-income Latinos in rural communities with limited food options [14].

The Latino population is not monolithic [15]. Different subgroups, such as indigenous Mexican immigrants and Latinos in rural southern California or Puerto Ricans in the Northeast, may have varying diet and lifestyle experiences due to state-level policies and/or community support and organizing [16, 18]. Subpopulations of Latinos have their own unique health needs and barriers to healthcare and public health information; as such, interventions need to be tailored to the distinct needs of Latinos in geographically and socially diverse regions of the US [17].

In this study, we built on our prior work, Ancestral Recipes, which adapted the evidence-based MyPlate-based recipe cookbook for health literacy level, language (Spanish, Purépecha), and food access, and also incorporated dialogues on diabetes management and healthy eating choices [12]. The cookbook, “Ancestral Recipes: From My Grandma’s Kitchen to Yours” was designed by Latinos for Latinos and includes both traditional recipes from Mexican cuisine and adaptations of those recipes for immigrants in the US [19].

## Methods

### Study design and objectives

The analysis presented in this study is part of a larger pilot randomized wait-list controlled trial (RCT) to test the feasibility, acceptability, and preliminary efficacy of a dietary and lifestyle behavior change intervention (see Cheney and colleagues [20] for details on the study design of the trial, trial sample, and main findings). In this article, we report on the data collected from focus groups to assess the acceptability of the intervention among the focal population: to elicit participants’ experiences of being part of the program, their satisfaction with program material and activities, and the effects of program participation on dietary and lifestyle changes.

The UC Riverside Institutional Review Board (IRB) provided expedited approval for the human subjects protocol # 22–092 approving all study materials and data collection procedures. All participants completed a consent form before the start of the research. The study was funded by the National Institutes of Health (NIH)-National Cancer Institute, Award #5P20CA242620-03 and registered with clinicaltrails.gov, NIH National Library of Medicine.

### Setting

This research was conducted in the Eastern Coachella Valley (ECV), a rural desert region in Inland Southern California. Latinos in this region are both the largest ethnic group and have the highest prevalence of diabetes [21]. These health inequities are perpetuated by low socio-economic status, limited education, language barriers, and legal status (e.g., non-green card holders or citizens) [22]. The ECV is home to 88,000 residents, 97% of whom identify as Hispanic/Latino, and 30–50% of whom live below the federal poverty line [23]. This region is also home to the largest Purépecha (an indigenous group from Michoacán, México) community in the U.S. These communities experience a profound shortage of local healthcare services, which, in combination with cultural (e.g., language) and structural (e.g., legal status) barriers to healthcare access, limits utilization of preventative and primary care and pushes many to seek care across the US-Mexico border [24–26].

One example of preventive care, in the form of public health education and promotion, is the Eat, Move, Live! (EML!) program, an intervention designed by the City of Hope (https://www.cityofhope.org/research/beckman-research-institute/population-sciences/health-equities/coe/coe-research) to promote change through education, healthy eating, and physical activity to prevent and manage chronic disease. The original, English-EML! version is a 10-week intervention for health disparity populations delivered by health educators in person or virtually in a group-based setting with weekly sessions. The design of each session is: a 60-minute presentation on a health topic, a 30-minute cooking demonstration on a healthy recipe, and a 30-minute exercise activity. Across the 10 sessions the health topics vary and include: (1) MyPlate dietary guidelines, (2) sugary drinks, (3) nutrition labels, (4) obesity, (5) diabetes, (6) stress, (7) cholesterol and heart disease, (8) eating healthy with limited economic resources, (9) body image and mental health, and (10) reducing food waste. Recipes focus on plant-based meals with grains such as pastas and quinoa. Exercise classes include yoga, band exercises, and aerobic activity. EML! has shown to reduce barriers to engage in healthy eating and lifestyle change behaviors potentially reducing risk of chronic diseases through sustainable prevention methods in low-income, minority communities [27].

### Culturally adapted intervention: ¡Coma, Muévase y Viva!

**Fig. 1:**
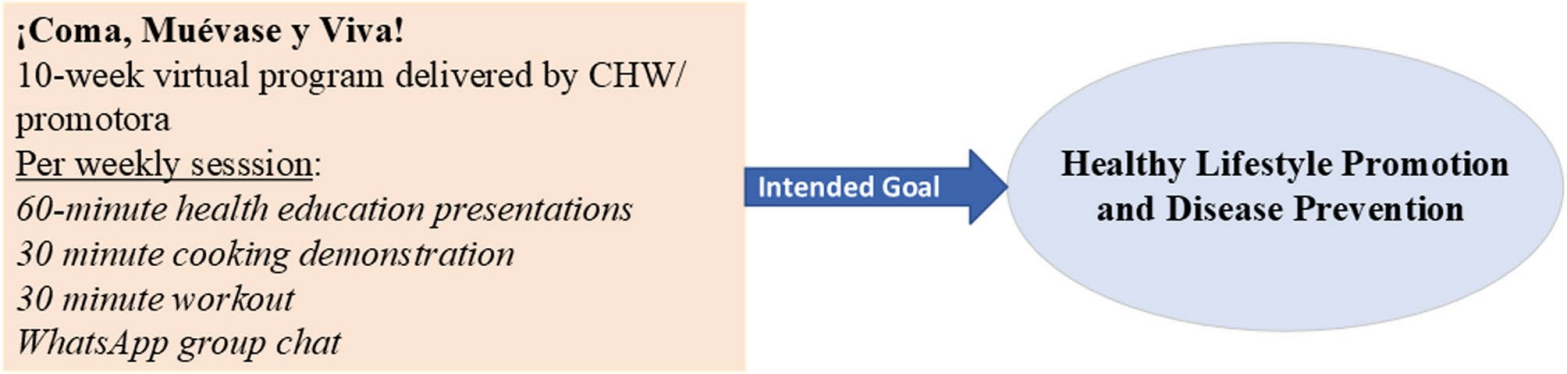
Conceptual model of intervention effects on chronic disease burden

The study team implemented an adapted version of the EML! program, ¡Coma, Muévase y Viva! (¡CMV!), tailored to the culture and language of the focal population and delivered by CHWs/promotoras. (For more information on the adaptation process, please see Cheney and colleagues work [20]). ¡CMV! is a 10-week program with weekly classes of up to 120 min. Fig. 1 provides a conceptual model of the intervention and its intended effects on healthy lifestyle promotion and disease prevention. Classes include a 60-minute presentation on a health education topic, a 30-minute cooking demonstration, and a 30-minute exercise activity. Our team, which includes bilingual (English, Spanish) faculty, staff, and students translated the original manual, presentations, and handouts into Spanish and worked with community health workers (CHWs/promotoras) to tailor the presentations and material to the target population in the ECV. CHWs/promotoras delivered the sessions, and weekly WhatsApp posts shared health education based on the weekly content, healthy recipes, and exercise ideas. Participants were encouraged to comment on the recipes and share anything related to healthy eating and exercise.

**Fig. 2:**
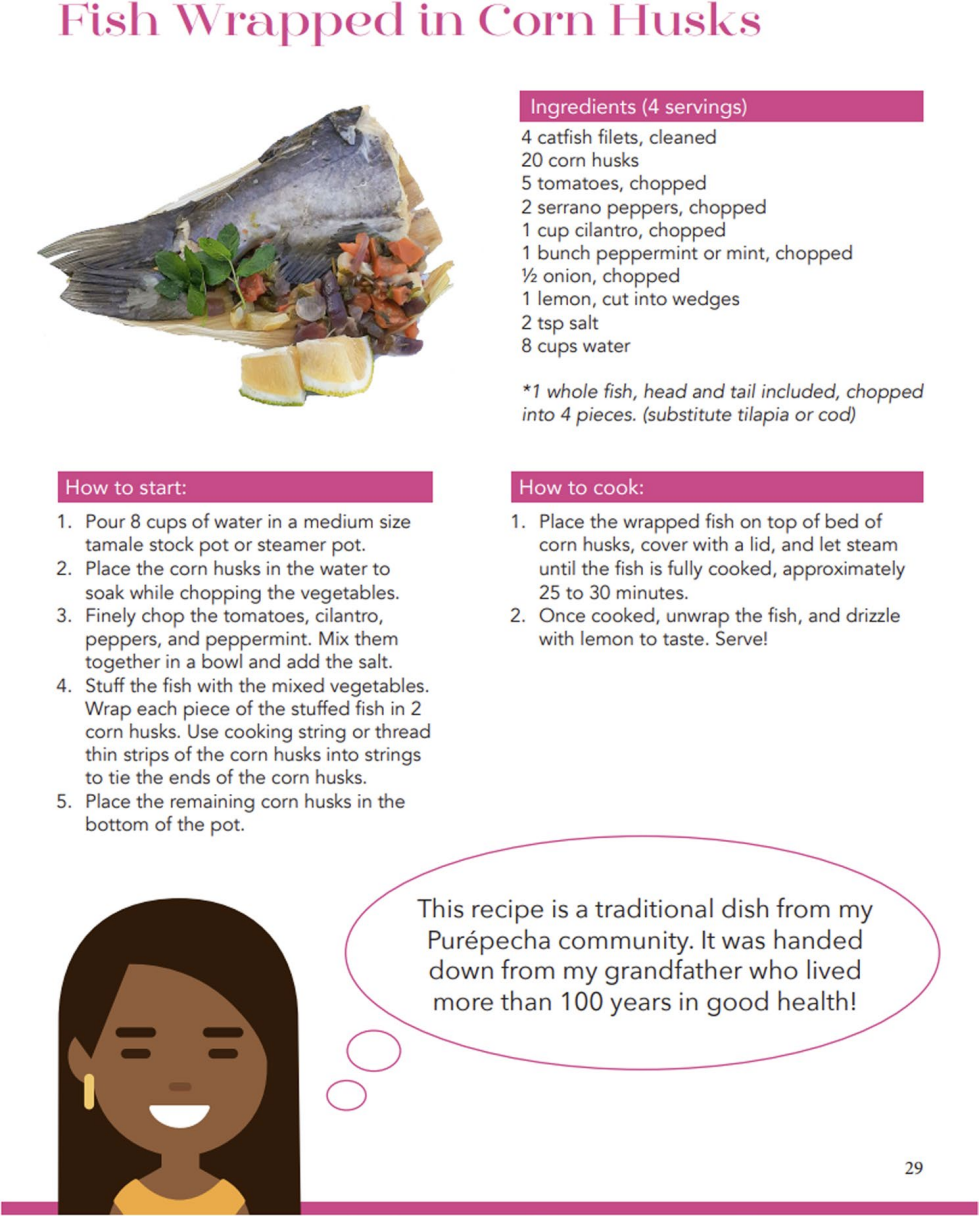
Recipe from Ancestral Recipes cookbook

Study team members simplified the Spanish translation and all presentations to make it more accessible to participants with limited health literacy and formal educatio. These changes included text reduction, images specific to low-income Latinx communities, and the inclusion of culturally specific elements such as pre-Hispanic food traditions involving the Three Sisters (corn, beans, and squash) using recipes from the Ancestral Recipes cookbook. Fig. 2 showcases one of the recipes used as part of the adaptation of the original curriculum.

The curriculum covered a range of health topics, including how to read nutrition labels (e.g., information on calories, sodium, saturated fat, total fat, and added sugars), managing and controlling diabetes (e.g., understanding risk factors), factors that contribute to obesity (e.g., visceral and subcutaneous fat), and complex understanding of cholesterol (e.g., differences between high- and low-density lipoproteins (HDL and LDL, respectively). Exercise activities were designed to be accessible to all participants and featured Spanish music familiar to the community. All content was modified to align with the program’s cultural theme, inspired by the Three Sisters depicted in the Ancestral Recipes cookbook.

### Study design

The qualitative analysis presented in this article is part of a pilot study involving an RCT of an adapted version of a diet and lifestyle change intervention. We report elsewhere the main quantitative findings for feasibility and acceptability from the pilot study and preliminary efficacy of the adapted intervention [20]. For the purposes of this article, we report on the qualitative evaluation of the feasibility and acceptability of the intervention with a subsample of study participants. We conducted observations of intervention sessions and focus groups with participants who received the intervention, including those randomized to the immediate intervention arm and those in the wait-list control group, who participated after trial completion. This approach allowed for obtaining the experiences and perspectives across participants in the initial and delayed delivery of the intervention.

### Recruitment and eligibility

We used purposive (nonrandom) and snowball sampling to recruit participants into the study [28, 29]. CHWs/promotoras recruited participants into the pilot study via outreach and study flyers throughout their networks (e.g., friends, family members, and local free clinic). Participants were eligible if they were: (1) 18 years of age or older, (2) lived in the rural desert region of Inland Southern California (e.g., Mecca, Thermal, Oasis, North Shore, Desert Shore, Salton City, Indio, and Coachella), (3) were able to engage in physical activity, (4) identified as Latina/Hispanic and/or Indigenous Latin American, (5) and spoke Spanish, English, or Purépecha. At the time of consent, participants indicated their interest in participating in a focus group to share their experiences and perceptions of the intervention. Of the 40 participants in the trial, 32 opted into the focus group at consent of whom all were invited to attend a post-intervention focus group. A total of 20, 16 from the initial intervention group and 4 the delayed intervention, attended one of three focus groups.

### Data collection

This study was carried out by the Unidas por Salud community academic partnership that is trilingual with abilities to carry out research in English, Spanish, and Purépecha. The partnership includes an academic investigator, community investigator, and CHWs/promotoras with expertise in human subjects research, research ethics, and community based participatory research approaches. The academic investigator, senior author (AMC) is a bilingual (English, Spanish) woman who holds her PhD in anthropology and expertise in qualitative research, led the qualitative evaluation. Trained bilingual research assistants, JM (woman), ABG (woman), and JC (man), conducted observations during the intervention sessions and of group chats. Two study team members, CHWs/promotoras (women), who hold expertise in focus groups facilitated the virtual focus groups with technical assistance from JM, ABG, and JC. Data collection was carried out in English, Spanish, and Purépecha depending on participant preference. Written material (consent form, socio-demographic surveys) was available in English and Spanish but not Purépecha. This is because Purépecha is primarily an oral rather than a written language and bilingual team members fluent in both Purépecha and Spanish provided oral interpretation for participants who preferred to communicate in Purépecha.

As part of the trial, the study team collected baseline data from all participants in person, by phone or Zoom. Observations of intervention sessions occurred online via Zoom and WhatsApp group chats and the post-intervention focus groups via Zoom. All focus groups were conducted in Spanish, audio recorded and transcribed in Spanish. Textual data (i.e., participant quotes) were translated from Spanish to English by bilingual team members. In this article, we primarily report on focus group findings on the acceptability of the intervention but also include observational findings to contextualize participants’ experiences and perceptions, as well as provide sociodemographic and health characteristics of the subset of focus group participants.

#### Focus group data

A total of three focus groups organized by group (initial or delayed intervention) were conducted. Two were held with participants in the initial group and one with the delayed group. We followed Guest and colleague’s standards for focus group data saturation with homogenous samples in which 80% of themes can be reached within 2–3 focus groups [30]. During focus groups, participants shared their perspectives on the ¡CMV! curriculum and their satisfaction with the program. Trained CHWs/promotoras conducted the group interviews using a semi-structured interview guide to gather information on several topics: experiences in the program, the program’s impact on knowledge of healthy lifestyles and chronic disease prevention and management, and its effects on perceived health status (see Supplementary file for the interview guide developed for this study). The questions were pilot tested, and wording was revised as needed. Focus groups lasted anywhere from 75 to 90 minutes and participants received a $25 gift card to thank them for their time.

#### Observational data

During weekly classes, study team members observed sessions and monitored WhatsApp group texts, taking detailed notes. Observations regarding participants’ motivation, engagement in the program, challenges encountered, confidence in making healthy lifestyle changes, and interactions in group chats were documented. We used observational data to contextualize themes identified in the focus groups, comparing the themes to team-level observations and comments via the group texts corroborating our interpretation.

#### Lifestyle behavior change goals

Our qualitative evaluation of the program's feasibility focused on lifestyle behavior change goals. At the beginning of the study, participants shared three lifestyle change goals they hoped to accomplish by the end of the 10-week program. These free-text responses were analyzed qualitatively to reduce items into categories and coded as eating healthy, maintaining weight, preventing chronic health outcomes, physical activity, cooking healthy, healthy family habits, and motivation for change. The post-test survey queried whether participants had completed the goal, were planning to complete it, or had no plans to complete it.

#### Sociodemographic data

Participants responded to socio-demographic questions on age, gender, race and ethnicity, indigeneity, country of origin, primary language, English proficiency, educational attainment, employment, number of household members, number of children, marital status, annual household, and health insurance. Participants reported on their use of food dispensaries assed by the question: "In the last month, how many times have you been to a dispensary to pick up food?" While not a validated measure of food security, this question captured access to food items commonly dispersed in the local food bank. Body mass index (BMI) was also assessed using objective measures of height and weight and imputing these numbers into the National Heart, Lung, and Blood Institute BMI calculator to determine BMI category: underweight = < 18.5; normal weight = 18.5–24.9; overweight = 25–29.9.9; and obesity = 30 or greater (https://www.nhlbi.nih.gov/health/educational/lose_wt/BMI/bmi-m.htm). Participants also reported their perceptions of chronic disease burden within their family responding to the question: “Please tell us how these comorbidities of being overweight/obese are a problem for you or your family” rated on a three-point likert scale from not at all to a great deal. Chronic disease was defined to include obesity, diabetes, high blood pressure, asthma, cardiovascular disease, cancer, and alcohol use. A CHW/promotora or team member administered the survey using Qualtrics (version 2.51.0; Qualtrics, Provo, UT) and obtained height and weight measurements.

### Data analysis

Qualitative data from the focus groups were transcribed using the Word transcription tool and imported into MAXQDA, a qualitative software analysis program [31]. For each transcript, research assistants listened to the audio recording while reviewing the transcript to ensure accuracy. In instances where clarity was needed, research assistants worked with study team CHWs/promotoras to review and revise the transcripts. JM, JC, and AMC conducted an inductive analysis, utilizing a line-by-line reading of the transcripts, to analyze qualitative data with the primary goal of discovering patterns and themes in and across participants’ narratives [32]. As a first step, a codebook was developed by reading the transcripts various times during the open coding phase and establishing a set of codes, code definitions, and examples that served as a guide for the analysis process [33] (see Supplementary material for an overview of the coding structure). Team members met to review the initial codebook and discussed the code application and any discrepancies. Once agreement was reached, one team member completed in-vivo coding, applying all codes to text segments. Finally, axial coding was carried out to compare the different themes and identify relationships that emerged across focus groups. While participants did not provide feedback on the findings, study team CHWs/promotoras assisted with the data interpretation and observational data were used to contextualize and interpret emergent themes.

Quantitative data from the sociodemographic and health-related survey responses and lifestyle change goals were analyzed using descriptive statistics, calculating frequency, percentages, means, and standard deviation.

## Results

### Participant characteristics

A total of 20 women participated in the focus groups. Most participants (95%) identified as Latina/Hispanic and 5% identified as Purépecha. Spanish was the primary language for 80% of participants, and 80% were originally from Mexico or Central America. The average age of participants was 45, with a minimum age of 22 and a maximum age of 66. Half (50%) of all participants had less than a high school education and 50% reported a household income of less than $25,000; 40% were uninsured and 60% reported picking up food from the dispensary within the last month. Most participants (85%) were concerned about obesity and being overweight, 45% about diabetes, 20% about high blood pressure, 30% about asthma, 30% about cardiovascular disease, 10% about cancer, and 10% about alcohol abuse. Two-thirds of participants were obese and 20% overweight (Table 1).

**Table 1:** Demographic characteristics

	Participants (*N* = 20) n(%)
Age	
Mean (SD)	43.85 (10.7)
Median [Min, Max]	45.0 [22.0, 66.0]
Ethnic Origin	
Latinx/Hispanic	19(95)
Indigenous Latin American	1 (5)
Country of Origin	
USA	3 (15)
Mexico	14 (70)
El Salvador	2 (10)
Missing	1 (5)
Number of Household Members (continuous)	
Mean (SD)	4.47 (1.6)
Median [Min, Max]	5.00 [1.00, 7.00]
Missing Answer	1 (5)
Children in Home under the age of 18	
Yes	14 (70)
Number of Children(continuous)	
Mean (SD)	2.2 (0.83)
Median [Min, Max]	2.00 [1.00, 4.00]
Married	
Single	2 (10)
Married	16 (80)
Single but living with Partner	2 (10)
Language spoken at home	
English	1 (5)
Spanish	16 (80)
English and Spanish	3 (15)
Purépecha (only)	0 (0)
Education level	
Less than High School	10 (50)
High School graduate	5 (25)
High School graduate/Professional School	5 (25)
Household Income	
Less than $15,000	3 (15)
$15,000- $24,999	7 (35)
$25,000-$39,999	7 (35)
Above $40,000	3 (15)
Health Insurance	12 (60)
Food Dispensary Utilization	12 (60)
Goal 1	
Eat healthily	7 (35)
Physical activity	7 (35)
Cook healthy	5 (25)
Motivation	1 (5)
Goal 2	
Eat healthily	6 (30)
Maintain weight	1 (5)
Prevent chronic health conditions	2 (10)
Physical activity	5 (25)
Cook healthy	2 (10)
Motivation	2 (10)
Missing	2 (10)
Goal 3	
Eat healthily	3 (15)
Prevent chronic health conditions	3 (15)
Physical activity	3 (15)
Cook healthy	6 (30)
Healthy family habits	1 (5)
Motivation	1 (5)
Missing	3 (15)
Goal #1 completion	
Goal completed	5 (25)
Plan to complete goal	10 (50)
No plans yet	5 (25)
Perceived Chronic Disease Burden Concerns	**Pretest**
Obesity/Overweight	17 (85)
Diabetes	9(45)
High Blood Pressure	4(20)
Asthma/respiratory illness	6 (30)
Heart disease/Cholesterol/Stroke	6 (30)
Cancer	1(10)
Alcohol Use	1 (10)
BMI (Pretest)	
Overweight	4 (20)
Obese	14 (70)

*In some cases, the percentages do not add up to 100 due to missing data

Each participant created three goals they wanted to achieve. As mentioned above, open-text responses of goals were coded as: eating healthy, maintaining weight, preventing chronic health outcomes, physical activity, cooking healthy, healthy family habits, and motivation for change. Among focus group participants in the initial intervention group, 25% completed their first goal and 50% planned to complete their goal.

### Overview

**Fig. 3:**
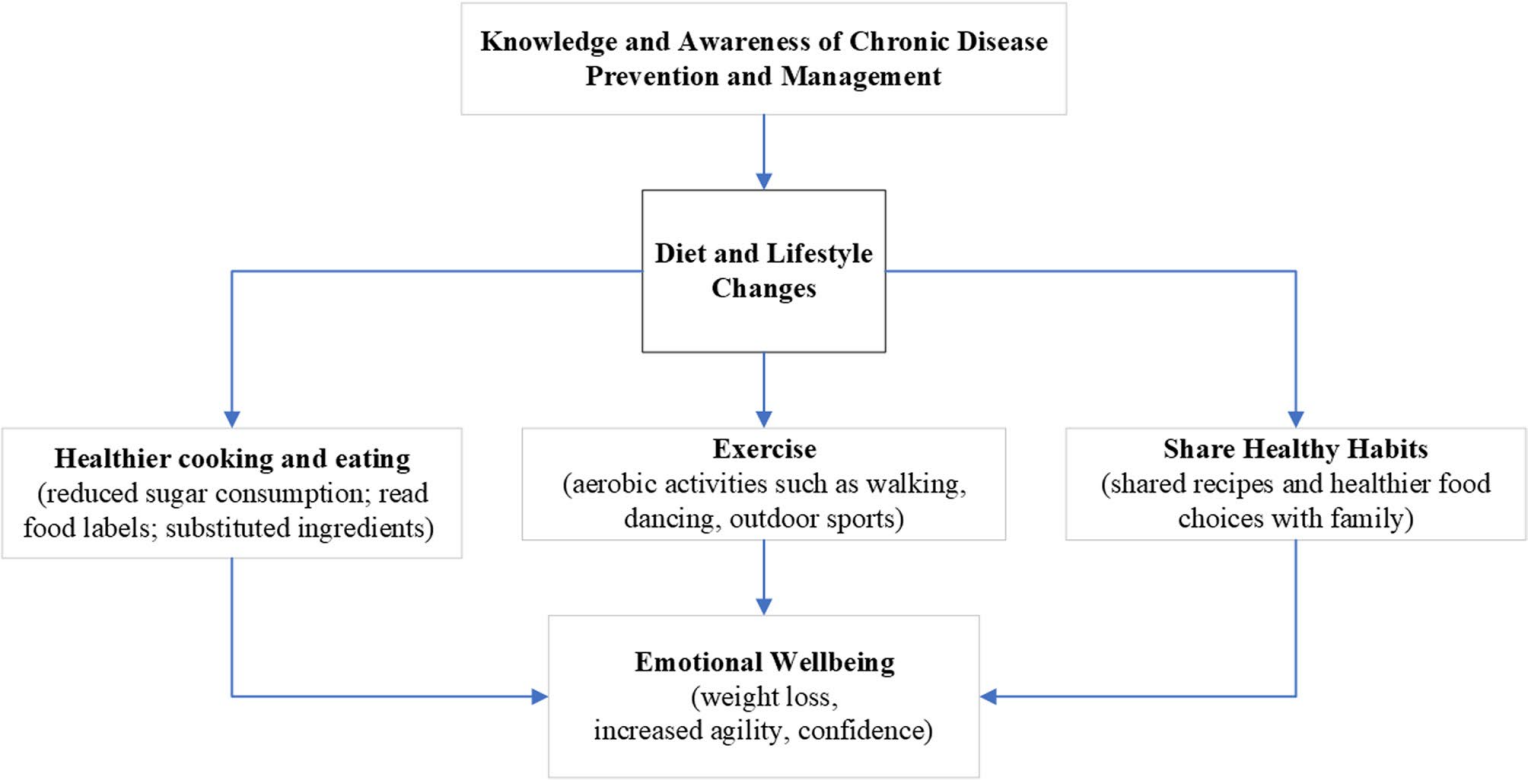
Conceptual model grounded in the inductive analysis of themes and their relationships

Our inductive analysis show that participation in the ¡CMV! program increased participants’ knowledge and awareness of healthy lifestyles and disease prevention motivating them to engage in diet and lifestyle change behaviors. Encouragement from the CHWs/promotoras who delivered the curriculum played an important role in behavior change. As this participant share: “I felt like they [the CHWs/promotoras] were encouraging us.” Participants reported changes in their diet, physical activity, and family routines along with outcomes such as weight loss, greater energy, increased emotional wellbeing (e.g., confidence), and improved ability to prevention and manage chronic diseases. Fig. 3 provides a conceptual model grounded in the emergent themes and their relationships.

In Table 2, we illustrate these relationships using excerpt quotes from focus group participants as evidence of the emergent patterns identified via the inductive analysis. Overall, participants shared how their participation in the program increased their knowledge and awareness of healthy lifestyles and chronic disease prevention. This motivated them to engage in diet and lifestyle changes, including healthier eating and exercise, which they shared with others, especially family. The following quote illustrates well the perceived impact of the program on diet and lifestyle change behaviors: “[The program] motivated us more to eat healthy, to exercise. Right now, I’m still doing these exercises, so now I feel like I’ve lost a few pounds, and I still have that motivation of eating healthy and exercising.” For some, program participation contributed to improvements in emotional wellbeing reducing depression and building confidence in behavior change.

**Table 2:** Participant perceptions of the effects of the ¡CMV! curriculum on diet and lifestyle changes and emotional wellbeing

Themes/subthemes	Participant Quote
Knowledge and Awareness of Chronic Disease Prevention and Management	
*Knowledge*	“I learned how to read product labels when you go to the store.” ~ FG1 P11
	“I learned you don’t need to use meat, and there are things that can be substituted.” ~ FG3 P6
	“I learned how to tell the difference in many things like what we talked about, sugary drinks. Often you think that because you drink a diet soda, it does not harm you, and does not have sugar.” ~ FG3 P5
	“I really liked the way we were able to avoid wasting food, freeze it, or give it to someone else, or use it right away.” ~ FG1 P5
	“I really liked learning about alternative ingredients for seasoning our food. It was something new that I learned and enjoyed.” ~ FG1 P10
	“Nutrition [classes] also helped me with my diabetes because I had to take pills in the morning and at night.. but right now it’s been more than a month that I haven’t taken a pill because my sugar is at a controlled level.” ~ FG2 P8
*Awareness*	“Realizing that fruits, it’s not just thinking that eating a fruit is healthy, without knowing that many of the fruits have a lot of sugar.” ~ FG3 P4
	“I liked [the program] a lot, because we learned so many things about the body, cholesterol, disease, and the heart, that maybe we were not aware of, and the program helps us to become aware.” ~ FG1 P10
	“I now look at the labels, the saturated fat, the two types of fat, how much salt it [food] has, how much sugar, and how many calories. In other words, the program made me very alert. And, I am happy to have joined the group and to see so many things that I had not even imagined. Now, if I eat three tortillas, I say: ‘Take out one and eat only two.’ I’m happy about this change and for the program.” ~ FG2 P8
	“The program created a little more awareness that I had to move. I had to walk...” ~ FG1 P9
Diet and Lifestyle Changes	
*Healthy cooking and eating*	“I liked that the recipes were easy to make and many of them were inexpensive. I think we are all looking for something healthy and affordable, and it was another way to learn to cook different things that are not in my routine, new recipes.”~ FG1 P10
	“What I really liked is that we made our recipe book a little bigger with healthy recipes for different tastes.” ~ FG1 P3
	“It’s something we needed because we’re always on the go and don’t have much time to spend preparing meals that take longer but are more nutritious.” ~ FG2 P6
*Exercise*	“I did like this program. In fact, I think I lost 8 pounds during the time I was doing the exercises.” ~ FG3 P2
	“Since I have a bad knee, I was doing the exercises the best I could, and I liked it, in the comfort of my home, and all that from the program, it was very good.” ~ FG2 P5
	“I really liked it. Thanks to the program I was motivated to walk . . . . The class motivated me because I didn’t walk at all. I have a son who says to me: ‘Mom, have you gone for a walk?’ He is also reminding me that I have to walk, and that motivates me. It motivated me to eat a little healthier. And my diabetes improved a lot.” ~ FG1 P9
	“I have always enjoyed exercising, but sometimes I feel lazy. Seeing the demonstrations motivated me to exercise every Monday and try to continue during the week.” ~ FG3 P8
	“I feel more energized now. Before, I would only do a little bit of exercise, but now I can do more because I feel lighter and more capable of exercising.” ~ FG1 P4
*Share healthy habits*	“My daughter said to me: 'You’re not going to cheat me [out of eating favorite dishes].' I did trick them. I made chorizo with egg, but it was soyrizo with egg. They even told me: ‘You put sausages in it.’.. I think I am going to integrate it [soy] back into my diet because I enjoyed it.” ~ FG3 P6
	“If we have good habits, then it’s easier for us and our family to follow it. But if we don’t make the changes, we can’t expect the family to do it.” ~ FG1 P5
	“.. you can eat healthy, not by eating so much junk food, but by the different varieties that you can make at home, with nutritious foods for yourself and your entire family.” ~ FG1 P3
Emotional Wellbeing	
	“I had serious depression because I wanted to learn how to eat healthier. Sometimes you read a recipe and think: ‘How do I start?’ But when you see the steps [in the program], because they are all very simple, everything seems very simple to me, which is a good thing. Because we are all in a hurry, we are tired. . . everything [in the program] didn’t take more than half an hour. I felt that it was very attractive, and everything seemed manageable to me . . . from the recipes to the exercises.” ~FG3 P6
	“I lost about 14 pounds from the time I started this program . . . I feel more at ease, calmer, more relaxed. Because I had already learned more about diabetes, that everything comes from being overweight.” ~ FG1 P5
	​“I could do the exercises slowly or in my own way, because it wasn’t like I had to follow a specific pattern in speed or strength or movement, and that gave me confidence.” ~ FG1 P3

### Knowledge and awareness of health and disease prevention

Seventy percent of participants in the study met the criteria for obesity based on their BMI and many shared how they or their family members had diabetes. Across the focus groups, participants discussed how participation in the ¡CMV! curriculum increased their health and nutrition knowledge and awareness of nutrition, how to prevent and manage chronic diseases, as well as awareness of the diet and lifestyle factors that contribute to chronic disease burden.

#### Knowledge

Participants appreciated learning about how to read nutrition labels, shop on a budget, avoid food waste, and recognize the potential harms of diet foods. They also appreciated gaining knowledge on the use of non-meat substitutes and alternative ingredients in commonly prepared recipes. Some participants commented on how knowledge gained via the health education classes helped them manage chronic diseases (e.g., monitor blood glucose levels).

#### Awareness

Participants commented on having gained a greater awareness of the contribution of diet and exercise to chronic disease burden. For instance, many commented on how the classes made them more aware of the high sugar content in many commonly eaten fruits, the relationship between cholesterol and disease, and the role of salt, sugar, and calories in poor health outcomes.

### Diet and lifestyle changes

The program encouraged participants to adopt healthy cooking and eating habits and engage in regular exercise such as walking and playing outdoors with their children. Participants discussed how the incorporation of diet and lifestyle change behaviors into their daily lives also influenced family wellbeing. Over two-thirds (70%) of participants were mothers of children 18 years or younger living at home. Their quotes illustrate how they prepared healthier foods for their families and sought to exercise while being with their family.

#### Healthy cooking and eating

As participants shared, the cooking demonstrations taught them how to prepare healthy recipes to cook at home. These recipes were well-suited to the participants’ needs, as they were straightforward to follow, considered time constraints for meal preparation, and encouraged use of food items (e.g., soybeans) commonly obtained from food dispensaries. These demonstrations also provided participants with alternative ingredients to use in their cooking, such as replacing salt with spices and other seasonings. Health and nutrition-based presentations discussed the importance of decreasing sweets and junk food from their diets and identifying ways to make healthier and more nutrition foods at home.

#### Exercise

Participants talked about how the weekly exercise demonstrations motivated them to become more physically active in their daily lives. Participants commented on the convenience of class exercises, which motivated them to maintain exercise routines throughout the week. Observations of the WhatsApp group texts indicate substantial interactions (posts, comments, likes) around physical activity. Participants shared pictures or videos of exercising such as walking, playing soccer with their family in the park, and running. Participants also reported improved health because of program participation. Across the focus groups, participants commented on how the program had a positive impact on their physical well-being, providing them with more energy, agility, and motivation to exercise regularly.

#### Share healthy habits

Participants discussed healthy eating at home and modeling ingredient substitutions. They encouraged their children and families to eat more fruits and vegetables, exercise regularly, and make healthier food choices. Family engagement in the program was important for participants and many shared how their family members (e.g., children) were aware of their efforts and provided reminders or encouragement to maintain diet and exercise changes. Among the participants who were mothers, they discussed the importance of preparing healthy meals for their families and teaching their children to eat healthier by eating less junk foods and more fruits and vegetables. They also discussed incorporating healthier food substitutes, like swapping sausage for soy, to encourage family involvement in healthier eating.

### Emotional wellbeing

Participants described adopting healthier eating and exercise routines, reporting weight loss, more energy, greater ability to be active, and increased confidence because of the ¡CMV! program. They also noted better control of chronic conditions such as diabetes, which they associated with feeling less concerned about their health and experiencing emotional benefits. After completing the program, participants commented on their ability to make healthier diet and lifestyle decisions, which increased their confidence in maintaining healthy routines and improved their emotional wellbeing. Many lost weight and felt better about the prevention and management of chronic diseases (e.g., obesity, diabetes). By making healthier eating choices and exercising more, participants experienced weight loss, which encouraged them to continue to make healthier diet and lifestyle choices even after the program ended. Some talked about having felt discouraged when trying to follow healthier recipes; yet, through the program they observed how to cook recipes that can be prepared and cooked within 30 min.

## Discussion

This study offers important insights into the role of CHWs/promotoras in adapting and delivering culturally tailored diet and lifestyle interventions. By drawing on the English-version EML! curriculum, CHWs/promotoras from the same community effectively adapted and implemented the Spanish-version ¡CMV! program, fostering trust and encouraging participation among Latina women in eating healthier and exercising more. Our work reinforces the importance of incorporating culturally relevant materials (e.g., recipes), engagement strategies (e.g., WhatsApp), and health education into diet and lifestyle change interventions to reduce chronic disease burden in health disparity populations [9, 10]. It also shows the value of culturally tailored interventions in meeting the unique needs of Latinx populations in rural communities, including linkages to food sources, resources to facilitate healthy eating and exercise, and strategies to incorporate diet and lifestyle changes into daily routines amidst structural and systemic constraints (e.g., low-wage labor, transportation) [11, 14].

Our qualitative evaluation of the ¡CMV! program indicated high acceptability among participants and that eating healthier and exercising more was feasible for study participants. The intervention also contributed to an increase in health knowledge and the development of skills to support healthy lifestyle changes and the prevention and management of chronic diseases. It also supported participants in sharing their knowledge with family members to promote broader diet and lifestyle changes within families. This is likely because the intervention addressed common socio-economic and cultural barriers to changes in diet and lifestyle.

Socioeconomic factors, including poverty and limited access to fresh fruits, vegetables, whole grains, and non-animal protein, increase risk for chronic disease burden among Latinx populations, particularly those in farm-working communities [10, 34]. Consistent with this, 60% of participants in our study reported utilizing food dispensaries and 90% were concerned with being overweight or obese. Culturally tailored programs, like ¡CMV!, address such inequities by collaborating with community food programs like Feeding America to identify ingredients accessible to low-income communities and incorporating healthy recipes that are affordable, easy to make, and align with cultural foodways [12]. The ¡CMV! program provides education on how to substitute cost-effective alternative food options (e.g., soy) often available via food dispensaries and low-cost food retailers to replace saturated fat food items like red meat. It also provides education on nutrition labels using culturally specific food items and discusses how to purchase fresh food ingredients on sale and conserve them. As others have shown, reducing economic and cultural barriers to healthy eating is fundamental to reducing chronic disease burden among low-income, racial/ethnic minority populations [35, 36].

Cultural and linguistic barriers may also increase risk for chronic disease burden among Latinos as they often have limited access to in-language public health nutrition education [37]. The ¡CMV!, program provided all instructions and materials in Spanish (participants’ preferred language) and oral translation in Purépecha as requested, which, we believe, may have increased the program’s effectiveness in supporting knowledge and skill acquisition. This effect was likely strengthened by accounting for participants’ prior education and health literacy levels, a phenomenon previously documented in the literature [38]. The cultural specificity of the study highlighted commonly consumed Mexican foods, such as tortillas and chorizo, provided education on their nutrition content and suggested healthy, accessible ingredient substitutions. These substitutions, along with new recipes from the Ancestral Recipes cookbook described above, promoted sustainable healthy eating habits with accessible, appealing foods [39]. The effectiveness of these targeted adaptations for Latinos in rural America paves the way for future programs tailored for the diversity of subgroups across the broader Latino community.

### Strengths and limitations

This study drew on multiple data sources that may have helped corroborate findings from participants’ qualitative quotes. For instance, field notes from weekly sessions and observations of WhatsApp group interactions provided content and reinforced themes identified in focus groups. These observational notes captured participant engagement, motivation, and challenges in real time, aligning closely with participants’ own accounts of their experiences shared during focus groups. Using multiple data sources strengthened the credibility of the study and offered a more nuanced understanding of how the intervention supported behavior change.

Despite these strengths, there are some limitations to consider when interpreting the findings. The sample self-selected into the focus groups, which may have introduced bias as those most satisfied with the intervention may be overrepresented in the focus group discussions. Another limitation of this study is the limited representation of participants in the delayed intervention group. Among the three groups, 16 participants from the initial intervention and only four from the delayed intervention group participated in the focus group discussions. We organized focus groups by group type (initial vs. delayed intervention) and only four participants in the delayed intervention participated in their assigned focus group, which does not meet the recommended size of 6 to 8 participants per focus group [40]. However, across focus groups, participants in both groups indicated high satisfaction with the intervention and its cultural relevance, feasibility in terms of healthy eating, food preparation, and physical activity. In our analysis we did not identify any meaningful differences in acceptability or feasibility by timing in intervention receipt (i.e., initial or delayed receipt).

## Conclusion

Our study findings highlight the need for a shift in public health and medicine to incorporate the voice of the community in the development and implementation of public health programs aimed at reducing chronic disease burden in health disparity populations. Growing evidence suggests that a one-size-fits-all approaches to health education and promotion are often less effective for supporting lifestyle and behavior change, particularly among historically marginalized groups who face cultural, socioeconomic, and/or geographic (e.g., rural settings) barriers limiting access to preventative health measures [41]. The implementation of culturally tailored intervention programs that meet the health literacy, language, and food access needs of low-income immigrant populations is critical to addressing chronic disease burden in this diverse health disparity population.

In our study, changes such as tailoring health literacy messages to include culturally relevant ways of accessing and consuming information (e.g., information shared by CHWs/promotoras, WhatsApp group chats, videos, pictures) and using language accessible to individuals with limited formal education appeared to play an important role in supporting participant engagement with the curriculum and program activities. This approach fostered a sense of empowerment and autonomy in managing chronic disease, instilling a sense of control via the acquisition of new knowledge and skills to support the well-being of themselves and their families. As we saw in our study, participants reported changes in diet, physical activity, and family routines, along with outcomes such as weight loss, greater energy, increased confidence, and improved chronic disease management. By adapting programs to the unique socio-cultural, linguistic, and economic needs of low-income immigrant populations, interventions like ¡CMV! can help address inequities in public health and healthcare systems that often limit access to prevention and education resources Providing accessible, culturally sensitive, public health education tailored to the unique needs of each community ensures that all individuals have the tools to make informed decisions about their health and well-being.

## Supplementary Information

Supplementary Material 1

Supplementary Material 2

## Data Availability

The datasets used and/or analyzed during the current study are available from the corresponding author on reasonable request.
