# Supplementary material for: ¡Coma, Muévase y Viva!: qualitative findings from a dietary and lifestyle change intervention for Latinas in the rural desert region of Inland Southern California
Source: BMC Public Health. 2025 Nov 19;25:4049. doi: 10.1186/s12889-025-25081-1 (PMC12628920; doi:10.1186/s12889-025-25081-1)
Supplement: Supplementary file 1 — Supplementary Material 1 [file 12889_2025_25081_MOESM1_ESM.docx]

**Introduction**

Thank you for taking the time to join us this evening. We've invited you all here to talk about the Eat, Move, Live program (Spanish name of program: Come, Muévase, y Vive). We'd like to talk about your thoughts about the nutrition and lifestyle education, the physical activity activities, and the cooking demonstrations/taste testing. We will also talk about your experiences in this program. Our conversation will be informal, but there are several questions we'd like to ask so I'll guide you along.

Before we start, let's go through some **ground rules**:

- Respect other’s point of view
- Be present and listen
- If you can, turn on your video camera
- What is said is confidential and stays in the room
- Remain honest

**Icebreaker**

Let's start with getting to know each other. Please tell us something about yourself. This could what you like to do or your favorite recipe or type of physical activity you like to do.

**Questions**

1. Let's talk about the Eat, Move, Live program you participated in. What did you think about the program?

- What were some of the things you liked about the program?
- What were some of the things you did not like or that could be improved?

1. What do you think about the nutrition and lifestyle information?
   - What did you like and dislike?
2. How about the physical activity exercises?
   - What did you like and dislike?
3. The cooking demonstrations/taste testing.
   - What did you like and dislike?
4. Now let’s talk about how well the program moves people toward living healthy lives. How can the program help people make nutrition and physical activity lifestyle changes? Feel free to use examples based on your own experiences.
   - Would you recommend other people in your family and community to participate in this program? Why or why not?
5. One of the last things we'd like to talk about is the design of the study. In this study, we used a design referred to as a wait-list control meaning that we had two groups: Group A and Group B. People were randomly placed in either Group A or Group B. Group A started first and then Group B 12 weeks later. What do you think about this approach?
   - What are the positives and negatives of using this approach?
   - Is this approach equitable? Why or why not?
6. Is there anything else you’d like to share about the Eat, Move, Live program?
