# Supplementary material for: ¡Coma, Muévase y Viva!: qualitative findings from a dietary and lifestyle change intervention for Latinas in the rural desert region of Inland Southern California
Source: BMC Public Health. 2025 Nov 19;25:4049. doi: 10.1186/s12889-025-25081-1 (PMC12628920; doi:10.1186/s12889-025-25081-1)
Supplement: Supplementary file 2 — Supplementary Material 2 [file 12889_2025_25081_MOESM2_ESM.docx]

**Supplementary Table 2**. Coding structure by theme and subtheme with definitions

| **Theme** | *Subtheme* | Definition |
| --- | --- | --- |
| **Curriculum feedback** | | |
|  | *Knowledge* | References to learning and understanding of health and related topics due to participation in the program. |
|  | *Awareness* | References to being aware, alert, better at recognizing health-related topics due to participation in the program. |
| **Diet and Lifestyle Changes** | | The general analytic category related to motivation to change diet and lifestyle behaviors that includes three subcategories: healthy eating, exercise, and share habits with family. |
|  | *Healthy Eating* | References to healthy recipes, ingredient substitutes (e.g., soy instead of red meat), reduced sugar consumption, increased consumption of fruits and vegetables. |
|  | *Exercise* | References to talking about wanting to exercise, engaging in physical activity (e.g., walking, weights, running around with kids), modifications to physical activities (e.g., bad knees). |
|  | *Share habits with family* | References to engaging family in diet and lifestyle change behaviors through family meal preparation or inclusion of children and family in physical activity and movement. |
| **Emotional Wellbeing** | | References to emotional and psychological wellbeing (e.g., feeling confident, anxious, depressed, at ease) before, during, and/or after participation in the intervention. |
